# Probability of regenerating a normal limb after bite injury in the Mexican axolotl (*Ambystoma mexicanum*)

**DOI:** 10.1002/reg2.17

**Published:** 2014-08-27

**Authors:** Sierra Thompson, Laura Muzinic, Christopher Muzinic, Matthew L. Niemiller, S. Randal Voss

**Affiliations:** ^1^ Department of Biology and Ambystoma Genetic Stock CenterUniversity of KentuckyLexingtonKY40506USA

**Keywords:** *Ambystoma*, axolotl, injury, limb, regeneration

## Abstract

Multiple factors are thought to cause limb abnormalities in amphibian populations by altering processes of limb development and regeneration. We examined adult and juvenile axolotls (*Ambystoma mexicanum*) in the *Ambystoma* Genetic Stock Center (AGSC) for limb and digit abnormalities to investigate the probability of normal regeneration after bite injury. We observed that 80% of larval salamanders show evidence of bite injury at the time of transition from group housing to solitary housing. Among 717 adult axolotls that were surveyed, which included solitary‐housed males and group‐housed females, approximately half presented abnormalities, including examples of extra or missing digits and limbs, fused digits, and digits growing from atypical anatomical positions. Bite injury probably explains these limb defects, and not abnormal development, because limbs with normal anatomy regenerated after performing rostral amputations. We infer that only 43% of AGSC larvae will present four anatomically normal looking adult limbs after incurring a bite injury. Our results show regeneration of normal limb anatomy to be less than perfect after bite injury.

## Introduction

Salamanders are renowned for their ability to regenerate limbs. This ability presumably originated hundreds of millions of years ago, perhaps tracing back to the evolution of tetrapod limbs. While we can only speculate about the origin of limb regeneration, it seems likely to have evolved in response to bite injury. Salamander larvae inflict conspecific bite injuries during early development while feeding within productive aquatic habitats that support high densities (Walls & Jaeger 1987; Semlitsch & Reichling 1989; Wildy et al. 2001). Such habitats also contain arthropod and fish predators that are capable of biting or grasping salamander limbs, thus causing injury (Gamradt & Kats 1996; Johnson et al. 2006; Bowerman et al. 2010). Larvae with damaged or missing limbs are less efficient at capturing prey and avoiding predators, and are susceptible to pathogens (Johnson et al. 2006). And, if a damaged limb were not repaired by regeneration, individuals could not efficiently disperse from aquatic larval habitats after metamorphosis and return for courtship and breeding. Thus, limb regeneration probably evolved because limbs are essential organs for completing a biphasic lifecycle, which is the ancestral lifecycle among salamanders.

It is interesting to compare bite injuries in nature to limb injuries that are staged in a laboratory. Typically in the laboratory, amphibians are anesthetized and limbs are amputated through the forearm or upper arm with a surgical knife. This yields a clean cut that causes the skin to retract and bone to protrude slightly beyond the amputation plane. Some researchers then perform a secondary surgery where they press gently on the limb to extend the bone beyond the amputation plane and then trim the bone (e.g., Knapp et al. 2013). Upon release of pressure, the bone retracts into the stump, creating a pocket that is more conducive to reepithelialization and wound healing. These procedures yield greater reproducibility of regeneration among individuals in experiments, ensuring that anatomically correct limbs reform in essentially every case.

Not all limb surgeries performed in the laboratory are simple amputation surgeries. For over a hundred years regenerative biologists have performed complex surgical manipulations to reveal the logic of tissue patterning during regeneration (Brunst 1961; Carlson 1975; French et al. 1976; Stocum 1978; Maden 1980; Bryant et al. 1981). If limb tissues are grafted in ways that juxtapose cells with different positional information, a range of abnormalities are observed, including missing or extra digits and limb elements. Examples of these different types of limb abnormality are also observed among amphibians from natural populations. Abnormalities observed in nature have been attributed to multiple factors, including toxicants, climate change, UVB, and pathogens (reviewed by Reeves et al. 2013). The majority of these factors are thought to cause abnormalities by disrupting limb development; however, there is growing evidence that limb abnormalities may be traced, at least in part, to how bite injuries disrupt limb regeneration (Sessions & Ruth 1990; Ballengée & Sessions 2009; Bowerman et al. 2010). To investigate this possibility further, it will be important to determine the probability for normal limb regeneration after bite injury. This probability is expected to be species specific and context dependent, and thus best measured in the field. However, it is difficult to control environmental variation and associate bite injuries with normal limb regeneration in natural populations.

In this study, we surveyed salamanders for limb abnormalities in the controlled laboratory environment of the *Ambystoma* Genetic Stock Center (AGSC). The AGSC maintains a captive bred population of over 700 *Ambystoma mexicanum* adults and several thousand juveniles and larvae. Adults are paired to generate clutches of embryos that are group housed until the time of hatching, which is approximately 20–25 days post fertilization. One week after hatching, approximately 50–100 larvae (approximately 1 cm in total length) from each clutch are group housed in the same bowl. During this group‐housing phase, larvae are susceptible to bite injuries from siblings that respond to conspecific movements and brine shrimp while feeding. The per bowl density of larvae is reduced over time by AGSC staff, so that after 80–90 days of growth, bowls typically have 10–15 4–5 cm larvae. Larvae within bowls are then separated and reared independently to generate stocks for users and to replace aging members of the adult breeding population. At approximately 18 months of age, females are moved into paired group housing while more aggressive males continue to be reared solitarily. Thus, the AGSC's husbandry methods, which have been in place for at least 30 years, rely on group and solitary housing to rear stocks. Here we report the results of a survey of limb abnormalities among larvae, males, and females. We detail types of limb abnormality and estimate the probability of normal limb regeneration after bite injury. We discuss the significance of our results for understanding frequencies of limb abnormality in laboratory and natural populations.

## Results

A diversity of limb and digit abnormalities (Fig. 1; Tables 1 and 2) were observed among axolotls in the AGSC. Abnormalities were documented for 2868 limbs from 717 adults. Approximately half of all adults presented four limbs with normal anatomy and full digits. In other words, approximately half of all adults have one or more abnormal limbs, with the former more frequently observed than the latter. Surprisingly, in 26 adults, all four limbs were abnormal. On a per limb basis, the most frequently observed abnormalities were syndactyly, ectrodactyly, and brachydactyly. While the frequency of ectrodactyly was similar between males and females, the frequency of syndactyly was approximately twice as high among males and brachydactyly was only observed among females. In general, males tended to exhibit higher frequencies of the more severe abnormality types (missing, extra, and backwards limbs).

**Figure 1:**
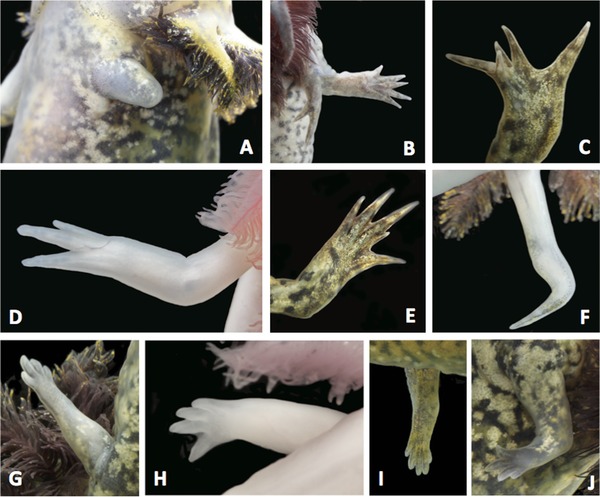
Abnormalities observed among adult *A. mexicanum* in the AGSC. Typically axolotls have four digits on forelimbs and five digits on hind limbs. (A) Axolotl with a small amount of limb tissue. (B) Axolotl with two forearms developing from the same upper limb element, and each has an atypical number of digits. (C) Axolotl with abnormal wrist and patterning of digits. (D) Axolotl with only three digits. (E) Axolotl with fused digits on hind limb. (F) Axolotl with a hook. (G) The nub limb pictured in Figure 1A after amputation and regeneration. (H) The limb with three digits pictured in 1D reformed a normal hand with four digits after amputation and regeneration. (I) The abnormal hind limb pictured in 1C reformed a normal foot after amputation and regeneration. (J) The forelimb with fused digits pictured in 1E reformed a normal hand after amputation and regeneration.

In comparison to adults, a higher frequency of larvae presented abnormal limbs. At the time larvae were transitioned from group to individual housing, only 20% were observed to have four anatomically normal looking limbs. Strikingly, all four limbs were observed to be abnormal for 47% of larvae. On a per limb basis, 65% of all limbs presented abnormalities consistent with a recent bite injury and not an abnormal regenerative response. For example, 68% of these abnormal limbs lacked upper arm or forearm elements, 25% lacked hands or feet, and the remainder exhibited missing digits. These results are consistent with larvae experiencing recent and recurrent bite injuries during the early group‐housing phase of husbandry.

The observation that larvae on average have more abnormal limbs than AGSC adults suggests that a high proportion of bite injuries incurred during early development are repaired by regeneration. To investigate this further, we related the observed frequency of limb abnormality in the larval phase to the observed frequency of adult males with four anatomically normal looking limbs. The estimate we derived is necessarily male specific because females are group housed in the AGSC and thus susceptible to recurrent bite injury and bouts of limb regeneration after the larval phase. Considering that 19.8% of larvae transition from group housing with four intact limbs, and assuming there is a 50:50 sex ratio, 9.9% of the 185 males (*N* = 18) with four normal limbs presumably never received a bite injury. Thus, we estimate that the probability that a male presents all normal limbs after incurring bite injuries during the larval phase is approximately 43% (185 – 18 = 167 males with four normal limbs that presumably did receive a bite injury divided by 401 − 18 = 383 males that received a bite injury). This frequency is much lower than the frequency of larvae observed with injured limbs. On a per limb basis, we note that the proportion of males with four abnormal limbs (3%) was dramatically lower than the proportion of larvae with four bitten limbs (47%). Also, the total number of abnormalities observed among males (*N* = 398) was small relative to the total number of limbs surveyed (*N* = 1604). These results suggest that the majority of limbs bitten during the larval period are repaired by regeneration, but even so the majority of adults present at least one limb abnormality.

**Table 1:** The number and frequency of limb abnormalities observed among AGSC larvae and adult *A. mexicanum*. For example, 185 males had four normal limbs

	Males		Females		Larvae	
	*N*	%	*N*	%	*N*	%
All limbs normal	185	46.0	160	50.6	50	19.8
One abnormal limb	129	32.2	67	21.2	27	10.7
Two abnormal limbs	62	15.5	49	15.5	20	7.9
Three abnormal limbs	15	3.7	27	8.5	37	14.6
Four abnormal limbs	10	2.5	13	4.1	119	47.0
Total	401		316		253	

**Table 2:** The number and frequency of limb abnormality types observed for larvae and adult AGSC *A. mexicanum*. For example, 145 limbs from males exhibited ectrodactyly

	Males		Females		Larvae	
	*N*	%	*N*	%	*N*	%
Ectrodactyly (missing digits)	145	36.4	106	30.7	29	4.4
Syndactyly (fusion)	138	34.7	57	16.5	–	–
Misplaced digits	40	10.1	37	10.7	–	–
Polydactyly (extra digits)	34	8.5	18	5.2	–	–
Ectromelia (missing limb)	31	7.8	10	2.9	458	70.3
Missing hands/no digits	–	–	–	–	184	28.2
Backwards elbows or hands	6	1.5	1	0.3	–	–
Polymelia (extra limbs)	4	1.0	1	0.3	–	–
Brachydactyly (partial digits)	0	0.0	115	33.3	–	–
Total	398		345		651	

Alternatively, it is possible that axolotl limb defects are caused by abnormal patterns of development and not bite injury. Indeed, a mutation associated with abnormal limb development is known for axolotls in the AGSC collection (*short‐toes*) (Humphrey 1967), and, more generally, inbred populations may express developmental anomalies as a result of inbreeding depression. To investigate this further, we amputated abnormal limbs in an attempt to induce regeneration and reform normal limb anatomy. We reasoned that limbs with abnormalities arising from bite injury might maintain the potential to reform a normal limb if an amputation was performed rostral to the anatomical position of the defect, in a region of the arm with normal anatomy. Conversely, limbs arising from abnormal development would not be expected to have appropriately patterned tissues to orchestrate regeneration, as is the case for the *short‐toes* mutant (Del Rio‐Tsonis et al. 1992). We amputated the limbs of eight axolotls: (1) three axolotls were missing almost the entire limb and only presented a small (∼3 mm) amount of upper arm tissue; (2) three axolotls were missing the hand or toes on the forelimb; (3) one axolotl presented a deformed wrist and hand; and (4) one axolotl presented fused digits on a hind limb. Only one of three axolotls with relatively little arm tissue regenerated (Fig. 1A, G). This suggests that a minimal amount of distal limb tissue is needed to initiate a regeneration response. For all of the other amputations, where defects were associated with the wrist and hand, amputation through the forelimb induced regeneration and formation of normal limb anatomy (Fig. 1H–J). These results show that abnormal limbs maintain the potential to self‐heal and reform anatomically normal structures. These findings support the idea that limb abnormalities in the AGSC are associated with bite injury.

## Discussion

In this study we observed and documented abnormalities from a captive population of *A. mexicanum*. Approximately 50% of adult *A. mexicanum* presented one or more limb abnormalities. The frequency of limb abnormality type varied between males and females. This most likely reflects differences in the way males and females are reared in the AGSC. After an early phase where larvae are group housed, larvae are separated into independent containers until sexual maturity is reached. At this time, females are housed in pairs. During this paired‐housing phase, females do not appear to inflict bite injuries of the type that engenders severe limb deformities. Indeed, more severe abnormalities were observed for males than females. However, paired females do damage each other's limbs. Brachydactly or missing digits was observed among 36% of females but was not observed among males. These injuries occur during feeding when one female attempts to suction‐capture a food pellet that is in close proximity to the other female's limbs.

It is interesting to consider if the high incidence of bite injury observed among AGSC axolotl larvae is similar to what occurs in natural amphibian populations. We observed that less than 20% of larvae complete the group‐housing phase of husbandry with four normal looking limbs. In the AGSC, axolotl larvae are provided live brine shrimp in excess. Larvae voraciously suction feed as they sense food items from nearby movements in the water. Larval densities are sufficiently high that food items and conspecific appendages occasionally co‐occur in the same area of the bowl. When this happens, limbs (and tails) are engulfed and damaged. In nature, bite injuries are thought to arise from aggressive interactions, when for example larger larvae attempt to prey and cannibalize smaller larvae (Johnson et al. 2006; Semlitsch & Reichling 1989; Sessions & Ruth 1990; Wildy et al. 2001). Indeed, we do observe that larger AGSC larvae will opportunistically cannibalize smaller cohort members. Field surveys of natural populations show that the incidence of bite injury varies greatly among salamander populations. For example, 15–80% of *Taricha torosa* (California newt) larvae within natural populations exhibited limb abnormalities indicative of injury (Johnson et al. 2001). The frequency of abnormalities observed among larvae in some natural populations of *A. macrodactylum* (long‐toed salamander) was estimated to be as high as 35% (Johnson et al. 2006). Furthermore, when *A. macrodactylum* larvae were paired in the laboratory for 48 h, abnormalities resulting from conspecific bite injuries were observed in 40% of encounters (Johnson et al. 2006). These estimates of limb abnormality are in line with estimates reported here for the AGSC when considering that bite injuries were allowed to accumulate among axolotl larvae during 2 months of group housing. Overall, moderate to high frequencies of limb abnormality are not atypical for natural and laboratory populations of salamander larvae.

Salamanders are generally regarded to be the champions of limb regeneration because they flawlessly and repeatedly regenerate limbs when precise amputation surgeries are performed on individuals. When an amputation surgery is performed, the cross‐sectioning lesion does not radically alter the positions of cells in the proximal stump, nor does it cause uneven loss of tissue from anterior, posterior, dorsal, or ventral domains. This creates an optimal environment for cells to communicate and function collectively to replace missing tissues via morphogenesis, intercalation, and patterning (French et al. 1976; Maden 1980; Bryant et al. 1981), although there remains debate about details of the underlying developmental mechanisms that pattern limbs during regeneration (McCusker & Gardiner 2013; Roensch et al. 2013). This scenario contrasts greatly with what happens when a scientist purposely changes the positions or types of cells in a regenerating limb, or when cell positions and cell numbers are altered as a function of bite injury. As an extreme example, when stump tissues are rotated and translocated to new positions and then an amputation is performed, this induces atypical and plural growth of digits and extremities (Carlson 1975). The tearing and mangling associated with a bite injury may similarly rearrange cells, and this alone, or in concert with subsequent bite injuries to the same limb, probably explains severe abnormalities observed in laboratory and natural populations. Our results show that if a sufficient amount of undamaged limb tissue is present rostral to a limb defect, a limb amputation can be performed to induce regeneration of a normal looking limb. We note that this potential has also been demonstrated for newts collected from natural populations (Tsonis & Eguchi 1985).

Our study shows that adults in the AGSC sustain bite injuries to limbs during development that are not perfectly repaired by regeneration. We speculate that some bite injuries so severely damage a limb that regeneration is not possible, even for the champion of vertebrate regeneration. High frequencies of abnormality are not observed among adults in natural populations because they are not afforded the protection of a laboratory environment. In the AGSC, adult salamanders with abnormal limbs ably perform as breeders, providing axolotl stocks to researchers around the world. Thus, it is not so important that regeneration yields perfect limbs; what matters is that the limbs or partial limbs are sufficient to achieve reproductive fitness (Tassava 2004). It is interesting in this regard to note that current AGSC husbandry methods select for reproductive fitness and this may be relaxing selection for regenerative potential. Understanding the relationship between reproductive fitness and regenerative ability, and understanding how inbreeding depression within small populations may affect regenerative ability (Williams et al. 2008), are important questions to pursue in future studies.

## Materials and methods

During the spring of 2013, a total of 253 group‐housed larvae and 717 group‐housed and solitary‐housed adult salamanders in the AGSC population were surveyed for limb and digit abnormalities. Limbs were considered abnormal if they presented any of the following: missing digits (ectrodactyly), partial digits (brachydactyly), fused digits (syndactyly), missing limbs or hands (ectromelia), extra digits (polydactyly), extra limbs or hands (polymelia), and digits protruding from the forearm or elbow. We note that in reviewing records where larvae are reared independently after hatching, which is provided as an AGSC service, the frequency of observing abnormal limbs is less than 0.5%.

Eight axolotls (6−9 cm snout‐vent length) that presented different limb abnormalities were anesthetized using 0.02% benzocaine and limbs were amputated with a sterile blade rostral to the limb defect. For defects of the hand/feet, limbs were amputated through the forearm or lower leg. For three individuals that only presented a small nub of upper arm tissue (∼3 mm), the distal tip was amputated. After surgery, axolotls were reared for 43 days and then limbs were photographed. The handling and surgical manipulation of axolotls was carried out according to University of Kentucky Animal Care and Use Guidelines.
